# “Too Much, Too Little, Too Late:” Exploring the Role of the Criminal Justice System Within the Narratives of Men Convicted of Sexual Recidivism in Norway and North America

**DOI:** 10.5964/sotrap.15759

**Published:** 2025-10-21

**Authors:** Ingeborg Jenssen Sandbukt, Danielle Arlanda Harris

**Affiliations:** 1 Division of Mental Health and Addiction, Oslo University Hospital, Oslo, Norway; 2 Department of Criminology and Sociology of Law, University of Oslo, Oslo, Norway; 3 Griffith Criminology Institute, Griffith University, Brisbane, QLD, Australia; 4 School of Criminology and Criminal Justice, Griffith University, Brisbane, QLD, Australia; University of Canterbury, Christchurch, New Zealand

**Keywords:** comparative analysis, qualitative research, quaternary prevention, recidivism, sexual offending

## Abstract

We present a targeted comparative analysis of two international samples of men incarcerated for sexual recidivism. A total of 30 men were interviewed: 16 from Norway and 14 from North America. The two jurisdictions from which these men were drawn represent the opposite ends of a global continuum of approaches to justice and punishment. We follow Rimer and Holt’s (2023) methodological approach and examine comparatively the way participants described their lived experience of custody, community release, and subsequent reoffending. Although all participants were convicted of crimes that carry the heaviest social stigma, the legislation and structural stigma to which they were subject were distinct. Many participants in both groups attributed their reoffending (at least in part) to systemic inadequacies of the criminal justice regime to which they were exposed. We discuss how these perceived inadequate responses of both countries appear in recidivism narratives in turn. First, we juxtapose the North American system (which was described as “too harsh”) with the comparatively relaxed Norwegian system (which was “too soft”). Second, we consider how treatment was offered (or mandated) in both jurisdictions and describe how there appears to be “no way out” of treatment for the North American men, and yet no way “into” treatment for the Norwegian men. Finally, we compare the men’s perspectives of being “set up to fail” with “too many hoops” in North America and “no safety net” in Norway. The relevance of their narratives for informing best practices is described and general policy implications within a framework of quaternary prevention are discussed.

## Abstract (plain language summary)

**Background:**

Although statistically rare, sexual reoffending is an international concern. Sexual offending carries clear and wide-ranging negative consequences, and its prevention is a consistent priority for criminal justice systems globally. Relatively little is known about the differential impact of international criminal justice systems on the lived experience of rehabilitation, release, and recidivism.

**Why was this study done?:**

We examined the justice system experiences of men convicted of sexual recidivism in North America and Norway. All our participants were convicted of crimes that carry the heaviest social stigma but the legislation and structural stigma to which they were subject were distinct. We compared two geographically different samples to offer insight into the most severe and complex of circumstances.

**What did the researchers do and find?:**

We thematically analyzed the lived experiences of 30 men (14 from North America and 16 from Norway) who were interviewed previously. We compared their recollections of custody, release, and recidivism. Men in both countries held the criminal justice system at least partially responsible for their reoffending. The North American system was described as “too harsh” with “no way out of treatment” and “too many hoops.” In comparison, Norway’s “too soft” approach provided “no way into treatment” and “no safety net” after release. Both systems were described as inadequate: North American men described being set up to fail by an overly-punitive system with too many restrictions and Norwegian men felt that short sentences, a lack of meaningful intervention, and meagre supports upon release provided little rehabilitation.

**What do these findings mean?:**

Our findings indicate that two very different systems were perceived to be equally ineffective in preventing recidivism. We recommend prioritizing interventions in which justice-involved individuals are able to participate meaningfully. Viewing our response to sexual offending through a quaternary prevention lens requires us to consider the unintended consequences of even well-meaning legislation and reminds us to switch course when it becomes clear that our policies and practices are causing more harm than good.

## Highlights

- The narratives and lived experiences of individuals convicted of repeated sexual offences are central to our understanding of recidivism prevention.
- Men in both Norway and North America attributed their sexual reoffending at least in part to the perceived inadequacies of their criminal justice system.
- Viewing our responses through a lens of quaternary prevention requires us to consider the unintended consequences of different criminal justice systems and approaches.
- We recommend an approach that holds people to account for their offences and acknowledges the harm they have caused while also providing intervention that allows meaningful reflection and reintegration into the community.

A sexual offence conviction carries substantial social stigma in most of the Western world. Norway and North America are certainly no exception, but vast cultural differences between these two nations moderate the extent to which individuals with sexual offence convictions are subject to certain legislative restrictions that result in structural stigma (Sandbukt, 2021). While the USA is known for its imposition of long sentences, extensive therapy requirements, and intrusive post-release restrictions (Harris & Levenson, 2021), Norway is instead recognised globally for its “exceptional” penal system which grants a second chance to *all* citizens who re-enter the community after a custodial sentence for *any* crime (Pratt, 2008; Ugelvik, 2016).

Although not yet the subject of a targeted comparison regarding sexual offending, these fundamental differences seem likely to impact the lived experience of custody, release and recidivism. This paper attends to this knowledge gap by presenting a cross-cultural comparison of such experiences for Norwegian and North American men convicted of repeated sexual offending. We present a targeted comparative analysis of two studies (in the style of Rimer & Holt, 2023). Hereafter, “Study 1” refers to the thematic analysis of archival files and transcripts from interviews with 14 men in two North American states and “Study 2” refers to the thematic analysis of the narratives provided by 16 Norwegian men currently serving custodial sentences in nine different prisons in five Norwegian counties.

## North America

The USA needs little introduction and due to a high concentration of popular law-and-order procedural television, their Criminal Justice System is relatively well-known internationally. With a population of more than 330 million, they are renowned for having the highest actual number of people in prison (almost two million) (WPB, 2024) and they are one of several countries who retains (and continues to practice) the death penalty. Since the early 1990’s (following the murders of Jacob Wetterling and Megan Kanka), Sex Offender Registration and Notification (SORN) legislation has proliferated across the country to the point where the names, addresses, photographs, and other identifying information is publicly available for more than one million citizens on a publicly available register (Harris & Levenson, 2021). Individuals convicted of sexual offences in the US are universally subject to draconian policies that restrict housing, employment, support systems, access to services, and physical safety. Interested readers are referred elsewhere for more detailed explanation and critique of the US experience (Harris & Levenson, 2021, 2022; Socia, 2014).

## Norway

Whereas North America incarcerates 664 people per 100,000, Norway’s current prison population rate is 55 per 100,000 (WPB, 2024). This small country (5.5 million) is one of three Scandinavian countries and five Nordic countries. Collectively, these nations are known as prominent examples of penal “exceptionalism” due to low imprisonment rates, relatively humane prison conditions, and the generally compassionate values and structures upon which their penal policies are built (Pratt, 2008). Despite broad critique of the reach and validity of the “exceptional thesis”—particularly from Nordic researchers during the 2000’s (see e.g. Barker, 2013; Dullum & Ugelvik, 2012; Smith & Ugelvik, 2017)—there is consensus among comparative criminologists that the Nordic countries are, in some ways, and relatively speaking, exceptional (Crewe et al., 2023).

The Norwegian Correctional Service’s guiding vision is “punishment that makes a difference”, underscoring their social mission as well as the positive consequences punishment shall have for the convicted individual (Kriminalomsorgsdirektoratet, 2021). Prisoners have the same rights as any other Norwegian citizen, including the right to vote, to free health care, and to higher education (Tønseth et al., 2019). Indeed, according to current correctional policy, a prison sentence is primarily described as an “opportunity” for rehabilitation and growth (Ugelvik, 2016). Sentences are relatively short, and imprisoned individuals have the possibility to apply for conditional release after two thirds of the sentence has been served (Ploeg, 2017). Common conditions during the parole period include refraining from alcohol and drugs and reporting regularly to a probation officer. Those who have committed sexual offences may also be prohibited from having contact with children. Moreover, upon conditional release from *forvaring* (indefinite preventive detention) individuals must notify their employers and landlords that they are subject to this regime. *Forvaring* implies a sentence of imprisonment for individuals who have committed a serious crime and who in the opinion of the court constitute a risk for reoffending (Kristoffersen, 2022). Release requires that there is no longer an imminent danger that the person will commit such a serious crime again, meaning that *forvaring* sentenced individuals can theoretically be imprisoned for the rest of their life (Appleton et al., 2025). However, when individuals are fully released (from *forvaring* or a determinate sentence), very few restrictions apply, even for those convicted of sexual offences. In fact, although certain jobs are unavailable for people with violent or sexual criminal records, Norwegian law ensures that a person’s criminal history is inaccessible to the public. These privacy protections are designed to facilitate reintegration and stand in stark contrast to the North American SORN legislation described above.

## Sexual Recidivism

Although sexual recidivism is statistically rare, it is doubtless an international concern. Sexual offending carries clear and wide-ranging negative consequences, and its prevention is rightly a consistent priority for criminal justice systems across the globe. Specific prevalence rates for sexual offending—however defined—are notoriously difficult to estimate due to the considerable proportion of crimes that remain undetected. Official recidivism rates are reported, measured and operationalized differently depending on jurisdiction, and their use for comparison between countries is therefore problematic (Yukhnenko et al., 2023). That being said, recidivism rates for individuals convicted of sexual offences are universally lower than for individuals convicted of other types of crime, and general recidivism is more common than sexual recidivism (Hanson & Bussière, 1998; Hanson et al., 2018; Lussier et al., 2023; Sandbukt et al., 2021; Yukhnenko et al., 2023).

## Treatment

The distinct criminal justice philosophies in Norway and North America are reflected in their respective approaches to rehabilitation of incarcerated individuals with sexual offence convictions. In North America, the focus is largely on risk management, and treatment is often mandated for longer periods, as was the case for the North American men included in this study (Harris, 2017). In contrast, rehabilitation is an explicitly stated aim of the correctional response in Norway, and therapeutic treatment options are voluntary. Access to treatment has traditionally been limited and is not always available to everyone who is motivated to engage. Recent changes have made offence-specific psychological treatment available to anyone assessed to be at a higher than average risk of reoffending. Thus, it must be noted that therapeutic experience varied among the Norwegian men in this study, with several men being involved in treatment for the first time during their current sentence (Sandbukt, 2024).

## The Present Study

This paper presents a targeted comparative thematic analysis of two independent studies from Norway and North America – representing what might be considered the ends of the penal continuum. The two studies were designed to address distinct research questions, but in this targeted comparison we focus our attention on: (a) the explanations of recidivism provided by the men, and; (b) the role that the system played in the men’s narratives. It is not our intention to “explain” recidivism in this paper or establish a causal relationship between system characteristics and reoffending. Rather, our objective was simply to describe the experience of custody and release for men in both jurisdictions, demonstrate how these experiences were interpreted by the men, and compare these perspectives in a thematic way. In doing so, we underscore that our goal is not to discover an objective truth, but rather to amplify the narratives (and explanations) of people who are rarely given a voice (Braun & Clarke, 2023).

We acknowledge the clear limitations of non-generalisability and sample bias. We have deliberately extracted individuals who likely pose a higher risk of reoffending sexually due to their persistent histories of detected recidivism and we argue that they constitute a population most urgently in need of formal intervention (Lussier & Frechette, 2022). The contribution of this study lies instead in the depth of detail that was provided during lengthy individual conversations with a very hard-to-reach population. This examination stands to provide useful insight into the most severe and complex of circumstances from which we can gain a deeper understanding of what members of this population believe they need from their respective criminal justice and support systems.

## Method

We follow Rimer and Holt’s (2023) approach and combine samples drawn from two separate projects in two different countries. Rimer and Holt (2023) conducted a targeted comparative analysis of two qualitative studies after combining the results toward one recurring topic (“addiction”). We combine results from two separate qualitative projects and samples in North America and Norway, and compare perspectives related to the topic “system inadequacy” in explanations of sexual recidivism. The participant recruitment and data collection processes for each study have been provided in more detail elsewhere and interested readers are referred to our previous work for more information (Harris, 2017; Sandbukt, 2024). Below, we briefly describe the participants in the current study before we describe the larger samples from which the present interviews were selected and outline our combined analytical approach of comparison through thematic analysis.

### Participants

The North American men (*n* = 14) were older at the time of interview (*M* = 57.75 years; Range = 49-75 years) and at the time of their most recent release (*M* = 36.75 years; Range = 20-53 years) than the Norwegian men (*n* = 16) (mean age at time of first interview = 46.3 years; Range = 30-66 years; mean age at last release = 35.75 years; Range = 26-48 years). The number of previous releases from prison (after sexual offence convictions) ranged from one to five (*M* = 2.5) for the North American men and from one to three (*M* = 1.6) for the Norwegians (based on self-report). We did not perform actuarial risk assessments, and the men were not subject to structured comparison in terms of other demographic variables. The 14 North American men were selected from a larger sample of more than 80 men who were interviewed about their process of release from custody and desistance from sexual offending. The men were interviewed on a single occasion either in custody (*n* = 8) or in the community (*n* = 6) and were asked to discuss their release and to account for why they returned to custody. The 16 Norwegian men were drawn from a larger sample of 23 men who had returned to prison for at least a second subsequent sexual offence. The men were interviewed twice in custody while serving their second, third, or fourth sentence for sexual offending. 13 of them were serving the indeterminate sentence *forvaring* (see Kristoffersen, 2022; Appleton et al., 2025) at the time of their interview.

### Inclusion Criteria

The main inclusion criterion was at least one known incident of sexual recidivism. Not all men who were interviewed and were known to have reoffended upon release were included in the present comparative study. The recidivism narratives herein were provided by those men who: (1) took at least some responsibility for their most recent offence/s (meaning that they did not deny having committed the offence/s), and; (2) were able to provide sufficient descriptions of their reoffending from which we could code our variables of interest.

### Exclusion Criteria

We define “recidivism” as the commission of at least one serious sexual offence which results in a period of incarceration, and which occurs after at least one previous serious sexual offence which also carried a custodial penalty. To be clear, we excluded several cases of “pseudo-recidivism”—where charges had been brought against someone after a period of custody, but for a historical incident that preceded their initial custodial sentence. These men spoke pointedly about the perceived injustice of serving a subsequent sentence after making therapeutic gains and leaving their offending behind them and thus had little to contribute to the question of “what went wrong during your most recent release?” Second, we excluded “undetected persistence”—in which someone admitted to having committed multiple offences against multiple victims but had only attracted the attention of the criminal justice system on a single (final) occasion. Third, we excluded any cases whose recidivism constituted exclusively minor or nonsexual “technical violations” such as “failing to register with law enforcement” or being “found in possession of a weapon” (e.g. pair of scissors).

### Procedure

Study 1 men were interviewed once each between 2011 and 2013 and semi-structured interviews were conducted individually using the Life Story Interview Protocol (Harris, 2017; Laws & Ward, 2011; Maruna, 2001; McAdams, 1993, 2008). The original study’s research questions were focused on understanding the men’s experience of release from custody and “desistance” (explained to participants as “successful community re-entry” and “not reoffending”). Thus, mentions of sexual recidivism usually emerged spontaneously in response to other questions such as “what was the low point of your life?” or “what has been your biggest challenge in life?” Interviews were digitally recorded, transcribed, coded, and re-experienced many times by the second author. Ethical approval for Study 1 was obtained through the appropriate university’s Institutional Review Board as well as in cooperation with the State’s Department of Corrections and partnering psychotherapeutic service providers. (Harris, 2017).

The Study 2 men were each interviewed twice in custody between 2021 and 2022 (Sandbukt, 2024). Inspired by Harris (2017), their semi-structured interviews also followed the Life Story Interview Protocol (Maruna, 2001; McAdams, 1993, 2008). Interviews were digitally recorded, lasted between 50 minutes and three hours, and the second interview followed within five months of the first. The advantages of conducting two interviews with each participant were many, but most importantly it facilitated a safe environment and sufficient time to cover the pre-defined topics while also enabling reflection in-between interviews. The original purpose of Study 2 was to focus explicitly on the process of recidivism, so an additional set of questions probed their prior releases and how they had ended up reoffending. The interview guide was used flexibly throughout both interviews and the first interviews were transcribed and studied before proceeding with the second interview. After transcribing the follow-up interviews, all interviews were coded according to Braun and Clarke’s (2006) steps of thematic analysis. As the study was exploratory, the data was approached with an inductive orientation. Ethical approval for Study 2 was obtained through the data protection officer at Oslo University Hospital, and data collection was approved by the Norwegian Correctional Service.

### Analytical Approach

Due to differential ethical approval and data access restrictions, the raw interview transcripts could not be shared between authors. Thus, the comparative analysis ultimately occurred through discussion of previous findings and was entirely post hoc. Previous thematic analysis of Study 1 participants identified the following themes: (1) “Relapse happened quickly”; (2) “Resources (especially money) upon release created a false sense of security”, and (3) “I wasn’t ready for release, but this time is different” (Harris, 2017). Previous thematic analysis of Study 2 participants established three overarching narratives that best accounted for their reoffending: (1) “Blaming the system”; (2) “Blaming their (hopeless) situation”, and (3) “Blaming oneself” (Sandbukt, 2024). The most prevalent themes across both samples suggested overwhelmingly that participants held the criminal justice system at least partially responsible for their recidivism. Thus, systemic inadequacies was a noteworthy theme discovered inductively. We followed Rimer and Holt’s (2023) approach to a targeted comparison and began by coding the interviews independently according to a set of predefined categories. First, the information was categorised according to time period (during incarceration, release planning, early release, and reoffending event). Then, a series of latent themes were developed to account for the experience of the respective systems to which the men were subject and how they narrated their subsequent offence. This interpretive coding process led to a more structural focus on perceptions of the justice systems in each nation and, in particular, the similarities and differences between their perceptions. This method was highly collaborative in that we met regularly to discuss preliminary findings and themes, but consistent with thematic analysis, our goal was never consensus or inter-rater reliability (Braun & Clarke, 2006). The range of jurisdictional differences, cultural idiosyncrasies, and subtleties of language helped justify our approach. Excerpts from the Norwegian transcripts were translated into English by the first author and edited for brevity and clarity by both authors in conversation.

## Results

The targeted comparison ultimately emphasised *how* the men spoke of the structures surrounding their sentencing and punishment in relation to their release and reoffending. Once the two sets of interview data were coded, discussed, and compared, several themes were clear. An overview of the guiding themes and some examples are provided in Table 1.

**Table 1:** Themes Identified From Study 1 (North America) and Study 2 (Norway) Transcripts

Theme	North America	Norway
1	The system was too harsh	The system was too soft
	The sentence was too long, treatment was too intense, registration was for life, treatment required payment	The sentence was too short/too lenient, no real intervention, no one to talk to, the offence wasn’t taken seriously
2	There’s no way out of treatment	There’s no way into treatment
	It goes forever, you’re never not at risk, you get convinced that you’re a monster, there’s always an assumption that you’ll reoffend – you’re dangerous forever, your risk is exaggerated	You don’t get help unless your sentence is long enough or you have reoffended, there’s an assumption that you don’t need treatment – risk is downplayed, and treatment is hard to access
3	Set up to fail – Too many hoops	Set up to fail – No safety net
	Unrealistic restrictions on movement, employment, residence, curfew, burdensome reporting requirements, unrealistic expectations – daily check-ins, weekly appointments, it’s impossible	No professional support, no proper aftercare or follow-up, no check in appointments or accountability, no treatment

We begin by discussing the similarities between the two sub-samples. Most of the men provided details of their reoffending with regret. They reflected on the difficulty of being asked to account for not just “why they did it” but how it had transpired that they had offended, gotten caught, been released, and then offended again. This invariably led to palpable guilt and shame:

Now, I’ve let society down, I’ve let myself down, the victims, all these girls. Twice, that’s enough. (Sven, Norway)

This sense of failure was clear when they were asked to describe the lowest point of their lives:

[The lowest point was] when I managed to fuck up for the third time and get *forvaring*. Absolutely. (Isak, Norway)

Failure was also clear in the US sample. While some of the men offered predictable or even clichéd descriptions of how good it felt to be released, Rudy was quick to change the tone when asked to describe “the best thing about getting out.” He paused and said:

Not to cast a pall over it, but there was nothing good about my release. Nothing. (Rudy, North America)

Below we discuss the perceived inadequate responses of both countries in turn. First, we juxtapose the North American system (which was described as “too harsh”) with the comparatively relaxed Norwegian system (which was “too soft”). Second, we consider the ways in which treatment is offered (or mandated) in both jurisdictions and describe how there appears to be “no way out” of treatment for the American men, and yet no way “into” treatment for the men in Norway. Finally, we compare the men’s perspectives of being set up to fail: with “too many hoops” (North America) and “no safety net” (Norway).

### North America Was “Too Harsh”

Brent articulated his view of the unfairly punitive nature of the juvenile justice system in his description of solitary confinement as a 16-year-old. He fully acknowledged his responsibility for the property and drug offences for which he had been sentenced, but attributes his subsequent commission of rape to the experience of sensory deprivation in custody:

When I was a juvenile, I was locked up and I was put in a cell in [State]…During that eight months I was going crazy. I had nothing to read, no one to talk to, I felt angry, I felt helpless, I felt like I was going crazy. I was really angry at my mother…They put me in this cell. I climbed the wall and escaped and raped a woman the next day. (Brent, North America)

Later in the interview (when asked about a turning point in his life) he again described his time in solitary:

The time in the cell changed me for the bad. I was a hyper 16-year-old with no books to read, no knowledge of when I’m going to get out. You just set me up to hurt and kill people or to hate people. It should have never happened. (Brent, North America)

Marshall provided another example of perceived injustice. After 11 years in prison for sexual assault, he successfully petitioned the court for graduated release and was subsequently found by a jury to be “no longer sexually dangerous.” Unfortunately, this process did not, in practice, translate to immediate community release as he (and his therapists) had expected. Instead, after he had satisfied state authorities that he had been “rehabilitated,” it was discovered that he had a “case hanging” in another jurisdiction and was required to complete a residual sentence in a maximum-security facility there, for a crime that had occurred many years earlier (before his first incarceration). This detection of pseudo-recidivism had the unfortunate consequence of undoing many of the social and relational gains Marshall had made while at the treatment centre. He entered the facility’s gradual release program in 1978, and for several years he left the property to work a full-time job and stay with his wife. In the final months of his penultimate sentence, he was away from the facility for several days at a time, returning only to attend a weekly therapy session. Marshall describes below what it was like to have the court (in his home state) declare him ready for release but then be transferred to prison in another jurisdiction, this time wearing the “sex offender” label.

Now this is part of the screwiness (sic) that happens. While I was out [on gradual release] I’ve bought a house, I’ve had a job, I’ve had two jobs at the same time, and I lose all that to go back to [name of prison]—one of the most dangerous places in the Commonwealth—to wait for parole. So, while I’m in there, all those animal instincts to protect yourself come back…I have done nothing wrong. I’ve gone to court and the court has said “you’re not dangerous no more, you can get put into society.” But because I haven’t done parole, I’m sent back to [name of prison]. With the statement that I’ve been to the treatment center…so I go back there, and I have to wait until the parole board sees me to get parole. And then finally I get parole, and this takes about a year to process, to get parole and to get released.

A therapist’s letter from his file provided a similar sentiment with an optimistic outlook:

Beyond any doubt, if [Marshall] did not have any underlying prison sentence, he would be fully ready for full release and able to assume a productive place in society as a functioning man, husband, and father. However, because of his remaining prison term, whatever the gains, he must return to prison to work towards eventual parole. He accepts the reality of this and the need to complete this last task of atonement for the crimes he has committed.

Although he had clearly made therapeutic gains during his graduated release, he still attributed some of the blame for his most recent offence (the murder of two women)—and the crime for which he is now serving a life sentence—on the counterproductive effects of that hanging sentence.

### Norway Was “Too Soft”

The Norwegian men were similarly frustrated by the criminal justice system and equally likely to hold it at least partially responsible for their recidivism, but for the opposite reason. Many Norwegian men considered their prior sentences to be woefully insufficient and somewhat useless from the perspective of correction or rehabilitation. Their greatest concern related to the impression that no one seemed to care much about the reason they were in prison. Several men shared the perspective that prison was best viewed as a form of warehousing that completely lacked any meaningful activities or intervention:

I guess I felt that it was more like a form of storage. That’s how I felt, I think. I guess that personally, I didn’t really experience it as a serious punishment. (Fredrik, Norway)

The thing was that the [most recent] prison stay [for his previous offence] was two months at an open facility. And, not to say anything negative about that prison but it was kind of like a school camp for 12-year-olds. You were treated as if you were 12 years old. … I guess it was, in a way, a kind of confirmation that what I had done wasn’t that bad. I asked for help so I could see a psychologist there, but I was told that my sentence was too short. So they couldn’t help me with that. (William, Norway)

Richard looked back with certainty that his recidivism could have been avoided if his behaviour had been taken more seriously much sooner.

For me, having two convictions, and for others as well, I think that maybe if I had gotten help right away and gotten someone to talk to right away, then maybe the other things [more recent offending] wouldn’t have happened. I really think so. Because the experience I’ve had with the psychologist [here] … it has woken me up, y’know? And I think it would be better to get that earlier. Um, and I think one could have managed to stop it already back then. (Richard, Norway)

Like others, William lied to the other prisoners about why he was incarcerated. When he was released, he also lied to his friends outside, and was seldom challenged by the very people who might have been most likely to help him desist, and succeed:

What became the main problem for me was that my conviction didn’t really have any consequences. No one really notices two months in prison and [people] kind of [think] “that’s nothing,” y’know? And it wasn’t a big deal in the media, and it was kind of swept under the rug. (William, Norway)

William further shared that when he was released from prison, his offending escalated quickly (Sandbukt, 2024). He was asked if he, upon release, thought he was going to “get his life together.” He replied “No, rather the opposite,” and then he added “because this was just a small thing, this was nothing to worry about, so I could just continue [offending].” He explained that he received this message through the system’s response of both: (a) a short sentence and, (b) the absence of treatment or professionals to talk to. This attitude reappeared during his second interview when he said “[my offence] was minimized by me, the police, and the judiciary.”

Whereas North American men are typically held on remand while awaiting trial, their Norwegian counterparts are usually permitted to live in the community. Although this approach might initially appear to be “soft,” Adrian’s account below suggests that the experience of uncertainty is interpreted as anything but lenient (see also Laursen et al., 2019):

Oh, the lowest point, that must have been before I was imprisoned the last time. It was while I was out and working. The investigation took a year and a half, even though I fully confessed. So, I just waited for one and a half years while working, um, and I was pretty depressed. I drank a lot because I knew I was going to prison and that it would be like three years. So, I just filled in time – I went to work, then home, drank, sat at home…So, this time, when they got me, I asked them to keep me in custody until I got my conviction. Because walking around in a holding pattern for one and a half years and not being able to change jobs, [or] find a girlfriend…Yeah, I think that was the lowest point. (Adrian, Norway)

### No Way Out of Treatment in North America

Men subject to the North American model of treatment felt paralyzed. Once they had entered the system and were required to attend a treatment group there was often (and quite literally) no way out. A curious Catch-22 situation had evolved in which one had to acknowledge that their previous behaviour rendered them at risk to reoffend but in turn, by affirming their risk, they were conceding that they remained at risk. They could never *not* be at risk to reoffend because if they argued that they were no longer at risk, then they were assumed to not be taking treatment seriously and were therefore at risk to reoffend.

This difficult position was shared by men currently in prison, but also by those who now lived in the community. For example, Giovanni (below) was interviewed after his release. He served two separate sentences for repeated child sexual abuse. He reflected on the relentless attention to his offending while in custody and alluded to a contagion effect of being “in a room with so many deviants” that he believes could have contributed to his recidivism:

When I was in [prison], y’know, they beat you like a dead horse. Same bullshit over and over and over, um... It puts like a dent in your head: never, ever, ever, ever, ever again. (Giovanni, North America)

Rupert was interviewed in custody. He knew his chances of release were slim because his governing offence was more serious than his prior offences:

I don’t want to stir it up anymore, you know? In group we’ve talked about different parts of our lives and stuff, and it’s like, well, jeez doesn’t anybody want to move on? I mean all this bad stuff, don’t you want to put it behind ya? But [the group facilitator] doesn’t think that we should, we should bring it up and keep bringing it up. I don’t like doing that, but I have to. (Rupert, North America)

Relatedly, Brent is currently serving his fifth sentence and will likely never be released. He reflected on how his coping mechanisms have changed over time. Where he might once have discussed his feelings more candidly, he has since learned to conceal his feelings and to not say “too much” in group:

When I think about how drastically I hurt people, I find it hard to live with myself. I mean, don’t call mental health because I’m not suicidal, but sometimes it gets hard. I wouldn’t tell anyone if I felt that anyway, because they would put me in the hole and then they wouldn’t let my therapist talk to me in the hole. The one I have the rapport with, they’d make me talk to someone different. So we don’t tell [our therapists when we’re feeling particularly low], y’know? That’s something we talk about amongst ourselves [with the other inmates]. (Brent, North America)

### No Way Into Treatment in Norway

The Norwegian men expressed a distinct feeling of being “stuck” *without* treatment during their prison terms. While the US men felt there was no way *out of* treatment and often gave examples of what has been described as a treatment industrial complex, the Norwegian men, in contrast, expressed a need for treatment that was never met. For example, Adrian was serving his third sentence and was upset that he had not received any treatment or follow-up:

[at the time of his most recent release] I had been inside for two years. I had worked a bit in a kitchen. That’s basically what had happened in the meantime. So, you know, I hope that a little more will change this time. And if I get some kind of follow-up [after release], that would be nice. Maybe the [treatment] program [which he will soon attend] is sensible. I don’t really know what to expect of it but it’s going to be exciting to see. … I’ve tried without any follow-up and that didn’t go very well. Um, and what do I have to do to… do I need another conviction to get it? Then it’s going to be *forvaring*. (Adrian, Norway)

Many drew a clear link between the “relaxed” style of the previous interventions and their most recent reoffending (see also Sandbukt, 2024):

I sent an inquiry to see the nurse at the open prison and I got in there and I explained that I had been in therapy [prior to imprisonment] but that my psychologist had quit and I asked to be referred to [the same therapy/treatment centre] or something similar. And I was told that, first of all, [the nurse] said that it was too short a sentence, and it wasn’t serious enough to qualify for that, but I also got the impression that getting help was very difficult. (William, Norway)

Robert below lamented that treatment was not ultimately available to him until his *forvaring* sentence, which he saw as too little, too late.

For those who end up with *forvaring* in the end, it’s sort of [too late]. Things were kind of picked up [in the end], y’know. But it was way too late. I also think that if I had been caught much, much earlier, it could have spared the suffering of several victims as well. (Robert, Norway)

Mikael also felt that the help he was offered came much too late. He spoke with confidence about the gains he was making with his present therapist, but added that “the treatment services in Norwegian prison are way too poor. It’s ridiculous”. He shared regret for what could have been:

[Now] I’m on my way to learn how to open up, y’know? But it’s kind of a bit sad that that’s because of a program that I applied for several times in the past [prior sentence]. And I don’t want to speculate, like, had I come here earlier then maybe not… y’know? We don’t know that. But I do see what I’ve gotten out of everything that has happened here [in this prison unit], right? … If I had been here the last time, then maybe I wouldn’t be sitting here talking to you [about reoffending] today. (Mikael, Norway)

These men expressed a clear sense of regret that they did not receive treatment sooner. Of the men who *had* received treatment, many were critical about its value. Lukas, for example, was serving *forvaring*, after having been incarcerated on three separate occasions for previous sexual offences. He spoke about having seen a psychiatrist during a period of release about 15 years ago but did not consider it helpful at all.

It gave me nothing. It was like talking to my dad. I can’t really remember us talking much about my problems. But that was probably also about me being good at hiding them. And he, the treatment provider, wasn’t able to see it. And maybe he should have been tougher in terms of challenging me, y’know? (Lukas, Norway)

Subsequently, because he did not receive the help he needed, Lukas lacked the tools he needed to stay crime-free upon release. He conceded that “I can’t do it on my own” and was generally skeptical of the system’s ability to rehabilitate him:

Well, my prior convictions have obviously not helped, y’know? So, it’s difficult to think that punishment works, um, in the sense of having a preventive effect. But then, in a way, it’s also kind of very short sentences. (Lukas, Norway)

Sebastian had seen a therapist in prison during a prior sentence but wanted something more than medication. He hoped this time he would be offered some kind of psychological support to talk about his offending:

In the first session I had [with the psychiatrist] she wanted to push medication on me, but I said from the start “I won’t take medication. I don’t struggle to sleep, I don’t see spiders at night, I just want to talk to someone about things.” And then in the second appointment, she wanted us to do a visualization exercise. And then [afterwards] she said that the session went well. I left with no fucking understanding of what had actually happened. (Sebastian, Norway)

Although many men blamed their recidivism on the lack of therapeutic intervention, they simultaneously questioned whether they would have been receptive to help back then. Their current accounts were of course influenced by the fact that they had reoffended. For example, after explaining that he was not offered any kind of treatment or follow-up after his last sentence, Adrian was asked if he wished that someone had taken his offences more seriously back then. He replied:

Yeah, well, it turns out that there’s a need for it [therapeutic intervention] so… [*trails* off] But had anyone asked me when I was about to be released the last time, I might have said “no”. Now, I would say “yes” because now it has happened again, and it hasn’t gotten any better. So, now it seems like a great idea. (Adrian, Norway)

### “Set Up to Fail” in North America With “Too Many Hoops”

We are not the first to suggest that the post-release requirements for North American men convicted of serious sexual offences are burdensome and ineffective. Others have articulated at length the many and varied ways that SORN legislation can (at worst) cause more harm than good (Harris, et al., 2024), and at the very least, fail to deliver a safer society (Socia, 2014). A compelling theme that repeatedly appeared for the North American sample was a feeling of being set up to fail by the onerous conditions, restrictions, and requirements of lifetime registration and notification. For example, the financial burden, the social costs, and the restrictions on movement all work against a person trying to thrive upon release. The expenses that Marshall described below are for example, more than 30 years old:

[In 1991] I have to pay $120 a month to a parole officer. I have to pay $240 for a therapist. So I’m paying $360 a month upfront, just to be out! Never mind the mortgage payments, the car payments, the medical fees for [daughter] … (Marshall, North America)

Rudy similarly described the rigamarole to which he was subject where it felt like he was stuck playing a game he couldn’t win:

I had to go to the police station and then parole the next day and register as a level 3. I went to parole first and they said, “you have to go to police first” and they said, “no, go there first.” I felt like they were just looking for a way to catch me. (Rudy, North America)

Far from being encouraged to succeed, parole officers and the system in general were frequently described as being determined to catch them out:

All the barriers that they put in front of you, you’re designed to fail. (Samuel, North America)

The PO’s job is to put you back in jail. (Dean, North America)

### “Set Up to Fail” in Norway With “No Safety Net”

The US system was so restrictive it created an untenable existence in which crime was sometimes the only answer, or at least appeared to be the best option. In contrast, the Norwegian system was held equally responsible for sexual recidivism, but due to desperation of another kind – desperation for intervention. After release, Sven said he had tried to get therapy. He talked to his GP and an inquiry was sent to a treatment unit that, according to him, never replied. He also applied to a local psychiatrist but was rejected. With the benefit of hindsight, now he was clear about what he needed back then:

I should at least have had someone, at least someone professional to talk to. Because there wasn’t any. Of course, I applied to the municipality and here and there, but it wasn’t considered serious enough and [they said] I wasn’t ill and I wasn’t this and that. Of course, it affected me…Like I’ve said, I have applied for treatment and not gotten any. And I guess I’ve been a bit annoyed and disappointed by that… Even though what I’ve done has been tragic it was really a cry for help, including the last time. And I didn’t get any help when I was out. I got no treatment. I had no one to talk to. (Sven, Norway)

This experience was something Sven regretted and took very seriously (see also Sandbukt, 2024). He was clear that upon his next release, he needed professional helpers as well as friends he could talk to as a sort of safety net:

My greatest fear is that I’ll…That maybe half a year or a year will pass, and I’ll commit new offences, y’know? Then my life is over. So you have to think about it, think about how the future will be. You have to, because that is the experience I’ve had…And this time around, when I get out, I’m not going to be released all alone. I’ll have people around me. I will. I’ve said that. (Sven, Norway)

## Discussion

This study compared two geographically different samples of men convicted for repeated sexual offences. Interestingly, many of the participants in both groups attributed their reoffending (at least in part) to systemic inadequacies of the criminal justice regime to which they were exposed. Norwegian participants blamed the system for failing to take their offending seriously. They felt that short sentences, “no one to talk to”, few treatment options, and meagre supports upon release provided little or no rehabilitation. In short, they were denied the intervention they felt (or later realised) they needed. In stark contrast, North American men described being set up to fail by an extreme and onerous system with too many restrictions that over-punishes and over-intervenes, and from which they felt there was no exit. Whereas the North American men got *too much,* the Norwegian men got *too little,* and all lamented with regret that any useful interventions came *too late* to prevent their reoffending. The relevance of these men’s narratives is therefore related to how we can provide individuals convicted of (persistent) sexual offending with an overall correctional intervention they can respond to and learn from within their respective criminal justice systems.

### Practical Implications

Taken together, the findings of this study suggest a need to strike a balance that is “just right” — between North America’s “too hard” (retributive and draconian) approach and Norway’s “too soft” (benign, hands off) approach. More broadly, however, our results are perhaps most usefully presented within the context of quaternary prevention. Quaternary prevention emphasises the fundamental principle of healthcare (the Hippocratic Oath) to “first, do not harm” (Harris et al., 2024). Quaternary prevention addresses the negative consequences of counter measures and seeks to ameliorate the negative (and often unintended) harms caused by various interventions (McCartan & Kemshall, 2023). This framework “completes the cycle of prevention” (Gofrit et al., 2000) and urges us to reflect critically upon the actions we take in the primary, secondary, and tertiary prevention spheres. Quaternary prevention therefore provides a useful framework in which to consider the various inadequacies of our contemporary responses to sexual crime (Harris et al., 2024).

Although well-intentioned, many approaches to treatment and intervention (especially in the tertiary space, where punishment occurs) carry serious unintended consequences or “iatrogenic harms.” “Iatrogenic harms are those which are unintentionally caused by the diagnosis (or label), the practitioner, or the intervention itself and are observed in circumstances where our response to a condition (or event) has been shown to do more harm than good” (Harris et al., 2024, p. 2). In this regard, North America’s SORN legislation has been subject to substantial critique (Harris et al., 2024). Dynamic risk increases when one’s connections to society are irreparably severed by inordinate periods of incarceration and when one’s community re-entry features emotional dysregulation, social isolation, and maladaptive coping (Harris & Levenson, 2021). Our collective goals of community safety are severely undermined when already vulnerable people subject to SORN legislation are prevented from meeting their basic human needs of survival, sustenance, relationships, and self-actualization. The North American men in this study clearly expressed the view that the regime they experienced both while imprisoned and after release caused more harm than good, and that it did not prevent their reoffending.

Perhaps more interesting and novel, is our finding that Norway’s “hands off” approach was perceived by participants to be equally ineffective and harmful, in that it contributed to reoffending. Norway is internationally recognized for its “exceptional” correctional approach and a penal system built upon values of equality, personal growth, and inclusion upon release. This apparently benign system is clearly well intentioned and, particularly when contrasted with the US, seems like a promising alternative to help individuals change and live prosocial lives. Among the Norwegian men, however, our results indicate that the experience of “not having someone to talk to” was the greatest concern. The fact that their sentences were experienced as having little meaning is consistent with Ievins and Mjåland’s (2021) descriptions of a “formally inclusionary” yet “surprisingly laissez-faire” punishment for individuals serving sexual offence sentences in Norway. Our results thus suggest that an overall approach perceived as *under*-intervention may also be experienced as causing more harm than good. McCartan and Kemshall (2023) suggest that efforts to “do no harm” means avoiding interventions that actively undermine desistance processes. Acquiring and sustaining a new offence-free identity has been seen as intrinsic to desistance (Harris, 2017; Harris & Levenson, 2021; Maruna, 2001). Moreover, desistance is largely recognized as a social process (McNeill, 2014; Nugent & Schinkel, 2016). Although desistance can occur in the absence of particular interventions (Harris, 2017), it rarely happens in a vacuum, and feedback, responses and acknowledgement from others have been found to help individuals who have sexually offended reflect upon their crimes and move forward (Kruse, 2020). The Norwegian approach is certainly not designed to undermine the process of desistance, rather the opposite. In exemplary ways, Norway’s system actively seeks to integrate people convicted of sexual offences both in prisons and upon release. Our results suggest that the well-intentioned approach of treating all prisoners equally may fail when people miss out on opportunities to discuss and process their offending (see also Laursen & Mjåland, 2025). In this regard, we emphasize how “too little” intervention was described as ineffective and may deprive individuals of important opportunities to move forward.

Viewing our response to sexual offending and recidivism through a lens of quaternary prevention requires us to consider the unintended consequences of even well-meaning legislation and to be brave enough to switch course when it becomes clear that our policies and practices are causing more harm than good (Harris et al., 2024). The North American “solutions” that restrict people from employment, accommodation, and relationships render these measures *too much;* The Norwegian approaches that starve individuals of connection and treatment render these measures *too little;* The interventions that cannot begin until after one has been adjudicated and reincarcerated, or are considered ineffective rather than helpful, render these measures *too late.*

We recommend an approach that is effective *and* meaningful. Regardless of the nature of their offending, and at the most basic level, justice-involved populations need someone to talk to and engage with in regular reflection. Men at risk of sexual recidivism require a safe space to discuss and process their lived experience (including but not limited to their criminal behaviours) *and* a fair opportunity for reintegration. To that end, we recommend prioritizing efforts that combat (rather than amplify) stigma and that seek to provide appropriate interventions for those who have experienced repeated failures. These interventions should be the ones they are able to respond to and learn from in meaningful ways.

### Strengths and Limitations

Our study is not without limitations, and the most evident ones concern sample bias and generalisability. Evidently, our findings are restricted to the perspectives of men convicted of sexual recidivism and cannot necessarily be applied to individuals convicted of sexual offences in general. Moreover, although our two samples were not directly comparable in many ways, we argue that there is good reason to compare their perspectives in spite of cultural differences. Our contribution lies in focusing on the experiences of this hard-to-reach population who, as “recidivists”, constitute an important population most urgently in need of effective intervention. The men we interviewed represent the extreme end of the criminal continuum, where correctional resources are pushed to their limit. Their uniquely long-term experience with the criminal justice system allows us to closely study the approaches that were perceived to be helpful or harmful in these rarest of circumstances. This must be considered an important strength, as our approach can shed light on potential systemic gaps that might not be apparent in lower risk cases, illuminate earlier opportunities for intervention, and strengthen our overall approach to public safety and crime prevention.

It is important to note the difference in method between studies. Study 1 was initially designed to examine desistance from sexual offending in North America and Study 2 aimed specifically to investigate sexual recidivism in Norway. The themes of recidivism for Study 1 were thus slightly less detailed and the interviews from Study 2 contained much richer descriptions of the prerequisites and surroundings of the specific instances of offending. It is also important to consider the historical dimension of this study. First, the North American men were interviewed over a decade ago, and the Norwegian men were interviewed more recently. Second, some of the sentencing and release experiences described (particularly by the North American men) occurred decades ago and prior to the enactment of current SORN legislation. The legal landscape in the US has changed considerably, and results should therefore be interpreted with caution. Similarly, the therapeutic options available to Norwegian men convicted of sexual offences have also changed in recent years. The “too late” theme demonstrated how many of the Norwegian men described that they now (finally) had accessed the help and support they claimed to have needed during previous sentences. It is also important to note that because of increased sentencing levels for sexual offending, some of the Norwegian men who questioned the usefulness of prior short sentences might have received a longer sentence, were they convicted today rather than twenty years ago (see e.g. Jacobsen & Skilbrei, 2021; Ievins & Mjåland, 2021). However, to be clear, a trend towards longer sentences should not be unilaterally framed as “progress.” Ultimately, how that time is spent in custody is more important than the length of a sentence.

### A Note on Truth

The version of events that a suspect gives to a police investigator at the time of arrest is unlikely to be a factual and complete account of the offence. Likewise, the account provided by a defendant on the stand during trial is at best a curated and rehearsed version of what happened. It follows that the account later expressed (with the benefit of therapy and/or hindsight) during interviews might also be an edited version of actual events. Of course, we are under no illusion that the versions of events that were shared with us during interviews are entirely accurate, but there is reason to believe that—without the threat of prosecution or conviction—our participants were less defensive and thus more likely to be honest. We remind the reader that, as is the case with qualitative research, it is less of a concern that we do not know the objective truth; what matters is how they have come to narrate their return to prison, and for us to include the words that they use and the explanations they offer at the time and in the moment of their interviews (Braun & Clarke, 2023).

### Conclusion

In summary, our results could be interpreted as the expression of a need for a Goldilocks approach to sexual offending – one that strikes a balance that is neither too hard nor too soft. Perhaps some of the North American men would have benefited from the Norwegian approach and vice versa. Of course, we are unable to discern how our participants might have experienced the approaches on the opposite side of the Atlantic, but there was a clear relief among the Norwegian participants that they were not subject to the legislative restrictions so common in the United States:

Y’know, I don’t think it should be like in the US, that you have to go around and say, “Hi, my name is Mikael. I’m convicted”, y’know? Then I’ll rather stay here. Y’know? I wouldn’t have a life outside, because you’ll never be able to return to society, so we really can’t adapt to those policies… In my opinion, if you’ve served your time and got out, you’re kind of done with it. And luckily many people in Norway think like that. (Mikael, Norway)

We also noted clear defeat expressed by the North Americans that their lives had been made irreparably worse by the heavy hand of punishment and exclusion.

Using a quaternary prevention framework, we have demonstrated the perceived perils of both over-intervention and under-intervention. Our participants described how North American punishment was experienced as stigmatizing and burdensome, and how Norway’s “light touch” approach lacked sufficient impact and may have undermined their desistance process. Ultimately, both strategies were viewed by the men we interviewed as having failed them. Nonetheless, we hope that our decision to give them a voice and share their experiences can inform best practices to reduce recidivism in the future.

## Data Availability

The interview transcripts from the current study are not publicly available due to confidentiality. Participants have not consented to share their transcripts beyond the research team.
